# The “LLQY” Motif on SARS-CoV-2 Spike Protein Affects S Incorporation into Virus Particles

**DOI:** 10.1128/jvi.01897-21

**Published:** 2022-03-23

**Authors:** Shouwen Du, Wang Xu, Yuhang Wang, Letian Li, Pengfei Hao, Mingyao Tian, Maopeng Wang, Tiyuan Li, Shipin Wu, Quan Liu, Jieying Bai, Xiaoyun Qu, Ningyi Jin, Boping Zhou, Ming Liao, Chang Li

**Affiliations:** a Department of Infectious Diseases, The 2nd Clinical Medical College (Shenzhen People’s Hospital) of Jinan University, Shenzhen, China; b Key Laboratory of Livestock Disease Prevention of Guangdong Province, Scientific Observation and Experiment Station of Veterinary Drugs and Diagnostic Techniques of Guangdong Province, Ministry of Agriculture and Rural Affairs, Institute of Animal Health, Guangdong Academy of Agricultural Sciences, Guangzhou, China; c Research Unit of Key Technologies for Prevention and Control of Virus Zoonoses, Chinese Academy of Medical Sciences, Changchun Institute of Veterinary Medicine, Chinese Academy of Agricultural Sciences, Changchun, China; d Institute of Virology, Wenzhou University, Wenzhou, China; e Non-Human Primate Research Center, Institute of Molecular Medicine, Peking University, Beijing, China; f Key Laboratory of Zoonosis of Ministry of Agriculture, South China Agricultural University, Guangzhou, China; Loyola University Chicago

**Keywords:** SARS-CoV-2, spike protein, LLQY domain, protein synthesis and cleavage, virus assembly

## Abstract

Severe acute respiratory syndrome coronavirus 2 (SARS-CoV-2) spike (S) glycoprotein mediates viral entry and membrane fusion. Its cleavage at S1/S2 and S2′ sites during the biosynthesis in virus producer cells and viral entry are critical for viral infection and transmission. In contrast, the biological significance of the junction region between both cleavage sites for S protein synthesis and function is less understood. By analyzing the conservation and structure of S protein, we found that intrachain contacts formed by the conserved tyrosine (Y) residue 756 (Y756) with three α-helices contribute to the spike’s conformational stability. When Y756 is mutated to an amino acid residue that can provide hydrogen bonds, S protein could be expressed as a cleaved form, but not *vice versa*. Also, the L753 mutation linked to the Y756 hydrogen bond prevents the S protein from being cleaved. Y756 and L753 mutations alter S protein subcellular localization. Importantly, Y756 and L753 mutations are demonstrated to reduce the infectivity of the SARS-CoV-2 pseudoviruses by interfering with the incorporation of S protein into pseudovirus particles and causing the pseudoviruses to lose their sensitivity to neutralizing antibodies. Furthermore, both mutations affect the assembly and production of SARS-CoV-2 virus-like particles in cell culture. Together, our findings reveal for the first time a critical role for the conserved L753-LQ-Y756 motif between S1/S2 and S2′ cleavage sites in S protein synthesis and processing as well as virus assembly and infection.

**IMPORTANCE** The continuous emergence of SARS-CoV-2 variants such as the delta or lambda lineage caused the continuation of the COVID-19 epidemic and challenged the effectiveness of the existing vaccines. Logically, the spike (S) protein mutation has attracted much concern. However, the key amino acids in S protein for its structure and function are still not very clear. In this study, we discovered for the first time that the conserved residues Y756 and L753 at the junction between the S1/S2 and S2′ sites are very important, like the S2′ cleavage site R815, for the synthesis and processing of S protein such as protease cleavage, and that the mutations severely interfered with the incorporation of S protein into pseudotyped virus particles and SARS-CoV-2 virus-like particles. Consequently, we delineate the novel potential target for the design of broad-spectrum antiviral drugs in the future, especially in the emergence of SARS-CoV-2 variants.

## INTRODUCTION

The spike (S) protein of coronaviruses, such as severe acute respiratory syndrome coronavirus 2 (SARS-CoV-2), is a single-pass type I membrane protein and the major determinant of virus entry, host range, tissue tropism, pathogenesis, and virulence (1, 2). The binding of S protein with the cell surface receptor initiates virus entry into host cells. As a class I viral fusion protein, the S protein forms a homotrimer on the mature viral surface, which comprises two major functional subunits—the receptor-binding subunit S1 and the membrane fusion subunit S2 (3). Cryo-electron tomography of SARS-CoV-2 virions revealed that S proteins are randomly distributed on the surface as a prefusion conformation (4), consistent with the trimer structure full-length S protein or its ectodomain (5, 6). Although the exact entry process of SARS-CoV-2 is being deciphered, the interaction between the S1 subunit and angiotensin-converting enzyme 2 (ACE2) is known to mediate virus entry (7, 8). During SARS-CoV-2 entry, a dramatic structural rearrangement of S protein is triggered from a metastable prefusion conformation to a postfusion structure of S2, leading to the formation of a fusion pore and virus shelling (9–11).

**FIG 1:**
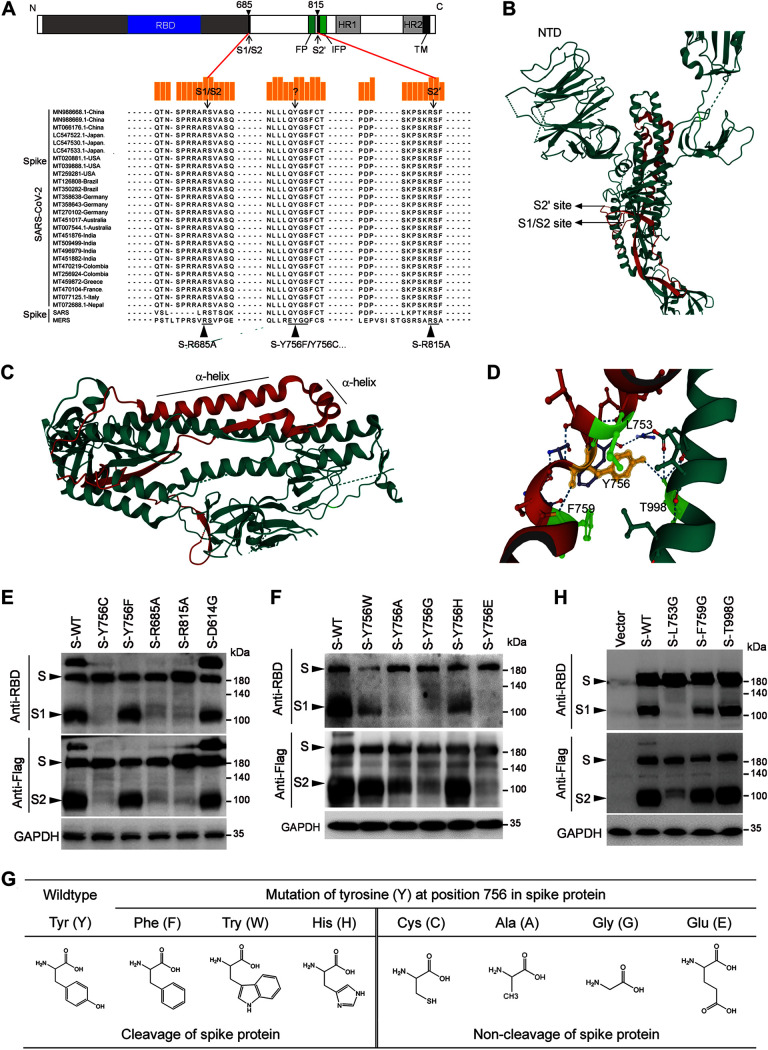
Tyrosine residue 756 is critical for the cleavage of the prefusion S protein. (A) Schematic of the domain structure of SARS-CoV-2 spike proteins and amino acid sequence alignment of the S1/S2 (R685) and S2′ (R815) cleavage site and the region between them in S2 subunit with the corresponding fragments of SARS-CoV and MERS-CoV. (B to D) The hydrogen bonds formed by Y756 and the leucine at position 753 (L753), the phenylalanine at position 759 (F759), or the threonine residue at position 998 (T998) in the folded S trimer can be observed in the cryo-EM structure of the S ectodomain trimer (PDB code 6VSB). (E) Western blot analysis of the effect of Y756, R685, or R815 mutation on the expression and processing of SARS-CoV-2 spike proteins using anti-RBD (S1) and Flag (C-terminal of S2). GAPDH was used as a loading control. (F) Western blot analysis of the effect of Y756 mutation to different amino acids on the synthesis and processing of S protein using anti-RBD and anti-Flag antibodies. GAPDH was used as a loading control. (G) A summary diagram of the regulation of S protein cleavage when Y756 is mutated to phenylalanine (F), tryptophan (W), histidine (H), cysteine (C), alanine (A), glycine (G), or glutamate (E). (H) Western blot analysis of the effect of L753, F759, or T998 mutation on the synthesis and processing of S protein using anti-RBD and anti-Flag antibodies. GAPDH was used as a loading control.

For SARS-CoV-2 and SARS-CoV entry, the proteolytic cleavage of coronavirus S protein by one or more host proteases, such as cysteine proteases cathepsin B and L or the transmembrane serine protease 2 (TMPRSS2), is essential to trigger membrane fusion (12, 13). TMPRSS2-mediated S cleavage at the S2′ site (residue R815 in SARS-CoV-2 [Fig. 1A]) must dissociate the S1 subunit and a cascade of refolding S2 subunit to postfusion conformation (5, 14). As a result, the TMPRSS2 inhibitors can be an effective candidate for clinical application against SARS-CoV-2 or other related viruses (15). In addition, unlike the SARS-CoV S protein, the SARS-CoV-2 S protein has evolved a polybasic cleavage site (RRAR) at the S1 and S2 boundary, which is effectively recognized by furin protease (11). Furin cleavage of S protein is required for its folding and maturation and putatively enhances SARS-CoV-2 infection and transmission or expands its tropism (16–18), while the cleavage at S1/S2 site during S synthesis is not essential for SARS-CoV-2 S-mediated viral entry (19). Additionally, the furin recognition site disappears during SARS-CoV-2 culture passage in Vero E6 cells (4). Although the mechanism is unclear, it may be related to the virus’s adaptability.

In structural analyses of the SARS-CoV-2, the S proteins were found to freely exist on the viral envelope, and the S head was connected to the viral membrane with the stalk domains with three flexible hinges in the S2 subunit (4, 20). Previous studies have shown that the S2 trimer presents a conservative symmetrical structure including the connector domain (CD), fusion peptide (FP), heptad repeat 1 (HR1), HR2, the transmembrane domain (TM), and cytoplasmic domain (CP), similar to the S proteins of SARS-CoV and Middle East respiratory syndrome coronavirus (MERS-CoV) (5, 18, 21). Among these, the FP, HR1, HR2, and TM domains are the key functional regions of the S2 subunit for the occurrence of virus-cell fusion and formation of fusion pore (22), but little is known about the function of the regions between the S1/S2 site and S2′ site for the prefusion conformation of S protein and its mediated virus assembly, egress, or infection. Therefore, some exploratory studies have been carried out on the two cleavage sites and the conserved regions between them for SARS-CoV-2 S-driven virus entry by single amino acid engineering mutation.

## RESULTS

### Y756 and its structurally linked amino acid residues are critical for the cleavage of the prefusion S protein.

The conservation analysis of S protein showed that the amino acid similarity among 27 SARS-CoV-2 isolates and SARS-CoV (CUHK-W1 strain) is 99.7 to 99.9%, and these SARS-CoV-2 strains share amino acid sequence similarities of only 74% with MERS virus (HCoV-EMC/2012) (Fig. S1A). Especially the region between the S1/S2 and S2′ cleavage sites of S protein (Fig. 1A), the amino acid sequences of these SARS-CoV-2 isolates and the SARS-CoV CUHK-W1 strain are completely consistent (Fig. S1B). Moreover, the structural analysis revealed that the linker fragment of two cleavage sites contains a couple of α-helices, which are connected via a flexible peptide, GS, forming a bend or turn (Fig. 1A to C) (6). Notably, the conserved tyrosine residue (Y) 756 at the downstream of the short α-helix is hydrogen bonded with the leucine at position 753 (L753), the phenylalanine at position 759 (F759), and the threonine residue at position 998 (T998) in the folded S trimer (PDB code 6VSB [Fig. 1D]), which is speculated to contribute to stabilizing the prefusion conformation of S protein for infection.

To investigate the influence of these amino acid residues between the S1/S2 and S2′ cleavage sites on the synthesis and function of S protein, a series of the S mutants was generated through site-directed mutagenesis (Fig. 1A), including replacing the arginine (R) residue 685 at the S1/S2 site or R815 at the S2′ site with alanine (A) and the mutation of Y756 to other amino acids such as cysteine (C) or phenylalanine (F). Then, the wild-type (WT) or mutant S protein with a C-terminal Flag tag was probed by Western bot analysis with the anti-receptor-binding domain (anti-RBD) antibody or anti-Flag antibody (Fig. 1E). We observed that SARS-CoV-2 S protein (WT) was processed into at least four forms with different molecular weights, i.e., monomeric S protein (observed at about 180 kDa), S multimers (>180 kDa), S1 (>100 kDa), and S2 (around 100 kDa) fragments in HEK293T cells (Fig. 1E), consistent with the previous studies (19, 22, 23). Furthermore, the natural variant of S protein with G614, which enhanced viral infectivity and transmissibility compared to D614 (24, 25), was processed similarly to the WT (Fig. 1E). As previously reported (19, 23), the R685A and R815A S mutants were produced as uncleaved forms (Fig. 1E). Of note, the mutation of Y756 codon to cysteine resulted in the expression of S proteins in the uncleaved forms, while the S proteins with the mutation of Y756 to phenylalanine could be cleaved (Fig. 1E).

To explore the regular pattern, we mutated the Y756 to other amino acid residues: tryptophan (W), alanine (A), glycine (G), histidine (H), or glutamate (E). We found that the mutation of Y756A, Y756G, or Y756E caused the S proteins to be produced as uncleaved forms, but the mutated S proteins with Y756W or Y756H were partially cleaved (Fig. 1F). Overall, the full-length S proteins with the aromatic amino acids, such as Y, W, or F, and the basic amino acids, such as H, were partially processed to the S1/S2 cleaved forms, and the other amino acid mutations could not cause the S proteins to be cleaved (Fig. 1G). Combined with its structural characteristics (Fig. 1B and D) (6), we speculated that the mutation of Y756 might affect the recognition of the cleavage sites on S protein and the corresponding proteolytic enzymes. For mutants with changes of Y756 to C, A, G, or E, the hydrogen bonds formed by tyrosine side chain disappear in the folded protein, which may not be conducive to the protein folding and conformational stability of S protein or even recognition of proteolytic enzymes. And then, three amino acids (L753, F759, and T998) related to Y756 were mutated to G. Western blot analysis showed that the L753G S mutant was largely uncleaved, and the F759G mutation significantly reduced the cleavage efficiency of S protein, while the T998G S protein was cleaved as effectively as WT S protein (Fig. 1H). These results indicated that besides R685 and R815 cleavage sites, Y756 and its hydrogen-bonded L753 and F759 are closely related to the spike cleavage efficiency in producer cells.

### Mutations of a structural element, 753-LLQY-756, reduce the infectivity of SARS-CoV-2.

**FIG 2:**
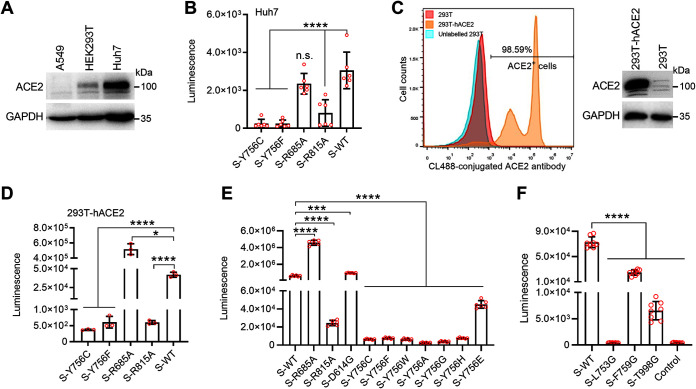
The mutation of Y756 or its structurally associated amino acid residues attenuates S protein-mediated viral entry by the pseudotyped viruses. (A) Western blot analysis of the constitutive expression of ACE2 in A549, HEK293, HEK293T or Huh7 cells and the overexpression of human ACE2 protein in the stable cell lines with anti-ACE2 antibody. GAPDH was used as a loading control for the cell lysate. (B) Lentiviral particles pseudotyped with SARS-CoV-2 S or its Y756C, Y756F, R685A, and R815A mutants were used to infect Huh7 cells; luciferase activity was quantified 48 h postinfection. (C) 293T cells expressing human ACE2 were constructed and identified by quantitative flow cytometry and Western blotting. (D) Lentiviral particles pseudotyped with SARS-CoV-2 S or its Y756C, Y756F, R685A, and R815A mutants were used to infect 293T-hACE2 cells, and the luciferase activity was quantified 48 h postinfection. (E and F) Lentiviruses pseudotyped with SARS-CoV-2 S or its R685A, R815A, D614G, Y756C, Y756F, Y756W, Y756A, Y756G, Y756H, and Y756E mutants, as well as L753G, F759G, and T998G mutants, were used to infect 293T-hACE2 cells, and the luciferase activity was quantified 48 h postinfection. Data are presented as means ± SD from 3 to 6 replicates. *, *P < *0.05; ***, *P < *0.001; ****, *P < *0.0001 (Student *t* test). n.s., not significant. Data are representative of those from three independent experiments.

We next evaluate the consequences of mutation of tyrosine 756 on SARS-CoV-2 infection using a lentivirus (HIV-1 NL4-3 strain) pseudotyping system (26). First, the constitutive expression of ACE2 in A549, HEK293, and Huh7 cells was identified in about 100 μg total cellular proteins by Western blotting. The results showed that ACE2 is highly expressed in Huh7 cells, followed by HEK293 cells, but is not found in A549 cells (Fig. 2A), indicating that Huh7 cells can be used to evaluate SARS-CoV-2 infection. Next, we packaged HIV-based SARS-CoV-2 pseudoviruses bearing the WT S and its mutants in HEK293T cells. The infectivity of these pseudotyped viruses was tested in Huh7 cells by the quantitative measurements of firefly luciferase levels. The results indicate that the R685A mutant SARS-CoV-2 pseudoviruses efficiently entered Huh7, similar to WT S (Fig. 2B). In contrast, Y756C, Y756F, or R815 mutation caused the S protein-mediated viral infectivity to be significantly weakened in Huh7 cells (Fig. 2B).

To further confirm pseudovirus infection, a HEK293T cell line stably expressing human ACE2 (293T-hACE2) was prepared through the lentiviral transduction system and blasticidin pressure selection. The ACE2-positive proportion of the 293T-ACE2 cell line was quantified as above 98% by flow cytometry, and the expression of ACE2 was detected by Western blotting (Fig. 2C). Then, the pseudotyped virus infection was performed on 293T-ACE2 cells. Consistent with the results with Huh7 cells (Fig. 2B), pseudotyped viruses with Y756 mutant spike exhibited extremely low infectivity (about 3- to 4-fold compared with that of the WT), similar to the R815A mutant, while R685A mutation resulted in higher infection mediated by spike protein than for WT or D614G S (Fig. 2D and E). More than that, very low infectivity or even no infectivity was also observed with Y756W, Y756A, Y756G, Y756H, or Y756E S-enveloped pseudotyped particles (Fig. 2E). These results imply that the S1/S2 cleavage of S protein during biosynthesis might not be the key factor for S-mediated virus entry, consistent with previous research (19, 23), while S2′ cleavage was necessary for S-mediated virus entry in our experiments. More importantly, Y756 may play an important role in SARS-CoV-2 spike-mediated infection by affecting its expression and processing.

In addition, we investigated the effect of L753, F759, or T998 mutation on SARS-CoV-2 spike-mediated virus infection. The three mutations significantly reduced the pseudovirus infection, especially the L753G mutation (Fig. 2F), consistent with the phenomenon caused by the Y756 or R815 mutation. Overall, some conservative residues 753-LLQY-756 in the connection region between the two cleavage sites, such as Y756 and L753, seem to play a critical role in SARS-CoV-2 infection.

### Y756 and L753 mutations alter the subcellular localization of S protein, similar to R815 mutation.

**FIG 3:**
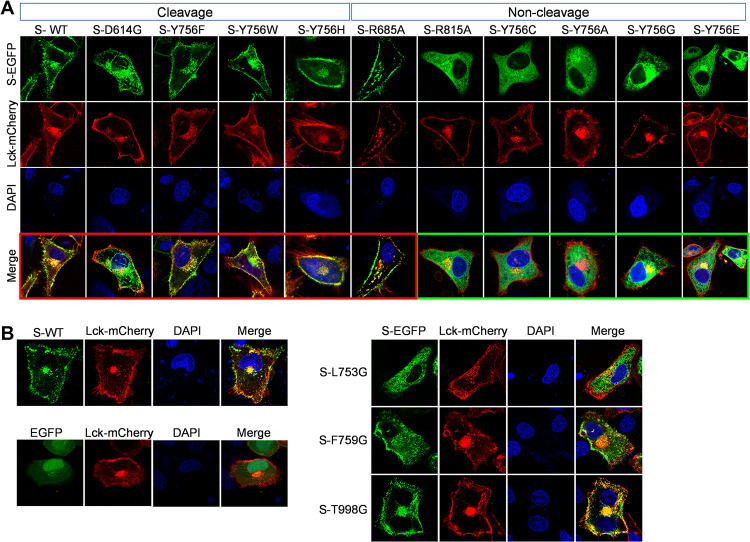
L753 and Y756 mutations, similar to R815 mutations, alter the subcellular localization of S protein. (A) Analysis of the subcellular localization of C-terminally EGFP-tagged S or its mutants in HeLa cells using a laser scanning confocal microscope (63×, oil). (B) Analysis of the subcellular localization of C-terminally EGFP-tagged S mutants (L753G, F759G, or T998G) in HeLa cells using a laser scanning confocal microscope (63×, oil). The tyrosine-protein kinase Lck was used as a subcellular localization marker.

Since these mutations, including R685, L753, Y756, or R815, affect the expression and processing of S protein, the subcellular localization of S protein may also be affected. For visual analysis of subcellular localization, the fusion expression plasmids of S or its mutants with a C-terminal enhanced green fluorescent protein (EGFP) tag and tyrosine protein kinase Lck with a C-terminal red fluorescent protein (RFP) tag were constructed and cotransfected into HeLa cells, and Lck was used as a subcellular localization marker; Lck localizes to the plasma membrane and pericentrosomal vesicles (27). As speculated, the subcellular localization of S and its mutations showed that the Y756F, Y756W, Y756H, and R685A mutant S proteins were processed and located at the cell membrane and near pericentrosomal vesicles, similar to WT S. Conversely, when S mutants were unable to be cleaved, the location of S proteins with Y756C, Y756A, Y756G, Y756E, and R815A mutations was observed to be an ambiguous subcellular location and diffusely distributed in the cytoplasm (Fig. 3A), indicating that Y756 mutation alters the subcellular distribution of uncleaved spike protein, similar to the outcome of R815 mutant S protein, although the mechanism may be different. In addition, the subcellular localization analysis of other S mutants showed that unlike for the F759G and T998G mutants or WT S, L753G mutation resulted in the irregular distribution of S protein after transient expression, whereas F759G or T998G mutant S protein colocalized with Lck on the cytoplasmic membrane (Fig. 3B). Overall, these data indicate that L753 and Y756 are of great significance to the correct processing of S protein, and the mutation of both may hinder virus packaging as a result.

### Y756 mutation attenuates S protein synthesis and the packaging of S-enveloped pseudotyped virus.

**FIG 4:**
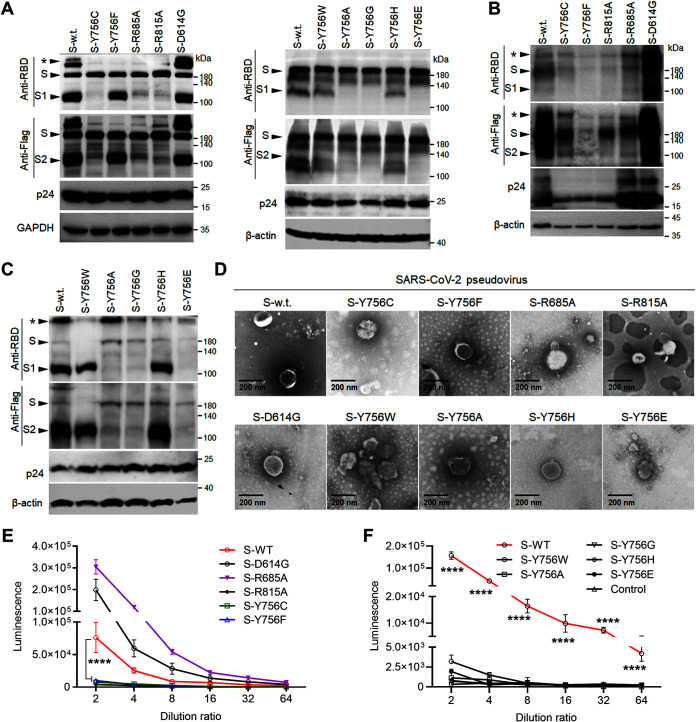
Y756 mutation attenuates S protein synthesis and the packaging of S-enveloped pseudotyped virus. (A) Western blot analysis of the expression and processing of S or its mutants in the pseudoviruses producer cells at 48 h after transfection with the indicated antibodies. HIV p24 and GAPDH were used as a loading control for the cell lysate. (B and C) Western blot analysis of the S protein or its mutants in ultracentrifuge pellets from cell culture supernatant with the indicated primary antibodies on the left blots. Uncleaved S and its cleaved S1or S2 products, as well as the HIV p24 and β-actin proteins, are also indicated on the left. The asterisk indicates S protein polymer. (D) Electron microscopy of the pseudotyped virus particles. The viral particles in supernatants were purified, concentrated, and added to 400-mesh copper grids covered with carbon-coated collodion film and then stained with phosphotungstic acid (1.0% [wt/vol]) for imaging using electron microscopy. (E and F) Infection analysis of pseudoviruses in 293T-hACE2 cells was performed by continuous gradient dilution and luciferase activity analysis. Data are presented as means ± SD from 6 replicates. ****, *P < *0.0001 (Student *t* test). Data are representative of those from three independent experiments.

Based on the results showing the effects of L753 and Y756 mutations on spike synthesis and processing in our experiments, we have boldly speculated that these mutations may affect the yield or quality of the SARS-CoV-2 pseudotyped viruses. During the packaging of S-enveloped pseudotyped lentiviruses in cell culture, the expression of S and its mutations was confirmed to be consistent with the data shown in Fig. 1E and F by Western blotting, and HIV p24 and glyceraldehyde-3-phosphate dehydrogenase (GAPDH) or β-actin were blotted as an experimental control (Fig. 4A). Also, in the pellet after ultracentrifugation of the pseudoviruses, the S proteins or their mutations on the surface of virus particles were evaluated by detecting the RBD or C-terminal Flag tag via Western blotting. Compared with the WT S or D614G S-enveloped pseudoviruses, the concentration of pseudoparticles carrying the Y756C, Y756F, or R815A mutation was significantly lower in the supernatants of 293T producer cells than the expression levels of uncleaved or cleaved S proteins and HIV p24 protein (Fig. 4B), suggesting that Y756C or Y756F and R815 mutations may not be conducive to the generation of S-coated pseudotyped virus. Interestingly, in the pellet of the pseudoviruses composed of Y756 mutant S proteins, Y756W or Y756H mutant S proteins were cleaved similarly to WT S, but Y756A, Y756G, or Y756E mutant S proteins on the surface of pseudoparticles were rare and uncleaved (Fig. 4C). The results showed that although both R685A S and R815A S were incorporated into pseudovirions in a noncleavable form, the transduction efficiency of the latter was lower than that of the former (Fig. 2D and E), suggesting that the S2′ cleavage of the S2 subunit was important for S-mediated pseudovirus entry into cells.

In addition, except that no pseudovirus particles bearing Y756G S were observed, the characteristics of the particles formed by the WT S or its other mutants were observed under a negative-staining electron microscope. The virions embedded by WT S or its mutants with Y756F, R685A, D614G, Y756A, or Y756H exhibited similar morphologies and a diameter of ∼200 nm, but the virions embedded by the Y756C, Y756W, Y756E, and R815A S mutants were irregular in shape (Fig. 4D). Amazingly, the spikes on the surfaces of the virions with R685A S were more prominent than that of the pseudovirions with D614 or G614 S (Fig. 4D), which may promote the S-mediated virus entry. Next, these pseudoviruses were titrated by 2-fold gradient dilution in 293T-ACE2 cells. Compared to the case with WT, D614G, or R685A S-incorporated pseudoviruses, regardless of whether the mutated S was cleaved, Y756 mutation caused S-assembled pseudotyped virus to lose infectivity (Fig. 4E and F), indicating that the Y756 mutation affected the S protein synthesis and processing and the packaging of its enveloped pseudovirions, which, in turn, affected S-mediated viral infection.

### Three site mutations structurally related to Y756 affect the production of pseudovirus with SARS-CoV-2 S protein, especially residue L753.

**FIG 5:**
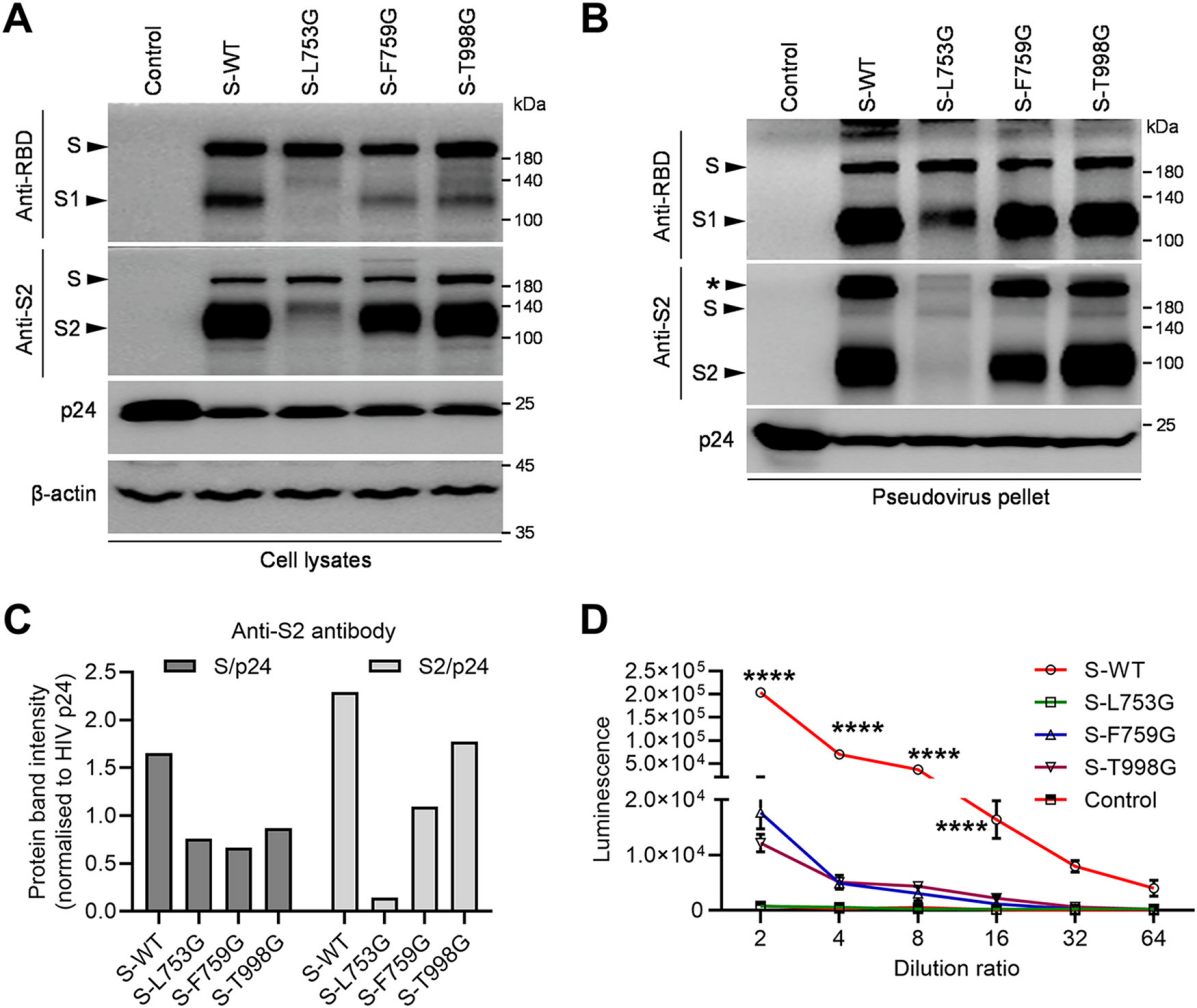
L753, F759, or T998 mutation interferes with the packaging of S-enveloped pseudotyped viruses. (A) Western blot analysis of the expression and processing of WT S or the L753G, F759G, or T998G mutant in the pseudoviruses producer cells at 48 h after transfection with the indicated antibodies. HIV p24 and β-actin were used as a loading control for the cell lysate. (B) Western blot analysis of the S protein or its mutants in ultracentrifuge pellets from cell culture supernatant by the indicated primary antibodies. (C) Relative quantification by grayscale scanning of the full-length S or cleaved S2 products from Western blot (B) with anti-S2 antibody. HIV p24 was used as a loading control. (D) Infection analysis of pseudoviruses with WT S or the L753G, F759G, or T998G mutant in 293T-hACE2 cells was performed by continuous gradient dilution and luciferase activity analysis. Data are presented as means ± SD from 6 replicates. ****, *P < *0.0001 (Student *t* test). Data are representative of those from three independent experiments.

We next evaluated the impact of the mutation of residue L753, F759, or T998, structurally related to Y756, on pseudovirus production with SARS-CoV-2 S protein. Pseudoviruses with each mutant S protein were produced, concentrated, and detected by Western blotting as described above. Similarly, the quantification of pseudovirus particles was performed using p24 protein as a reference. The expression of WT or mutant S protein was detected by anti-spike RBD or S2 antibody in the producer cells (Fig. 5A). For the spike on the surface of pseudoviruses, T998G S was efficiently cleaved into S1/S2 products, consistent with WT S, while F759G mutation reduced the amount of intact S or S1/S2 cleavage (Fig. 5B and C). Compared with the WT spike incorporated into pseudovirus particles, the L753 mutation significantly reduced the number of spikes and caused the mutant to be largely uncleaved (Fig. 5B and C). Overall, these data indicate that L753, F759, and T998, structurally related to Y756, affect pseudovirus production with SARS-CoV-2 S protein, especially residue L753.

### Mutations of the 753-LLQY-756 helix decrease the incorporation of S protein into SARS-CoV-2 virus-like particles.

**FIG 6:**
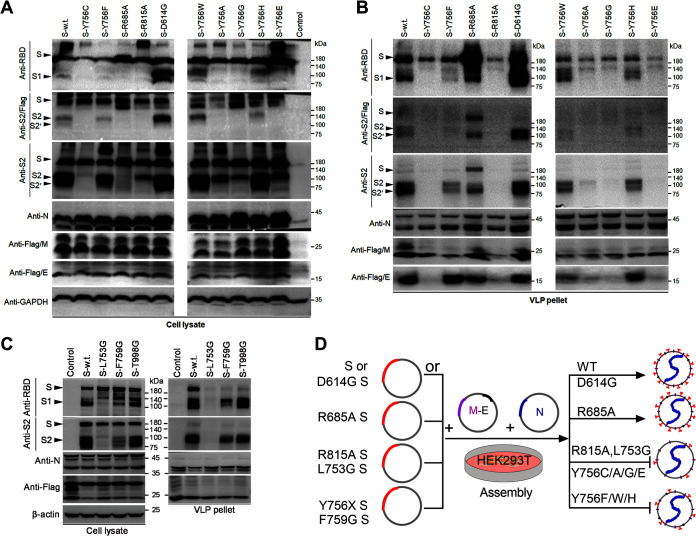
Y756 and its structurally associated residue mutations decrease the incorporation of S protein into SARS-CoV-2 virus-like particles. (A) Western blot analysis of individual expression of the S, M, E, and N proteins in the cell lysate at 72 h after transfection with the primary antibodies indicated on the left. GAPDH was used as a loading control for the cell lysate. (B) Western blot analysis of the S, M, E, and N proteins in ultracentrifuge pellets from cell culture supernatant with the primary antibodies indicated on the left. (C) Western blot analysis of the S, M, and N proteins in VLP producer cells (left) and ultracentrifuge pellets (right) from cell culture supernatant with the primary antibodies indicated on the left. Uncleaved S and its cleaved S1, S2, and S2′ products, and the N, M, and E proteins, are also indicated on the left. (D) Schematic outline of SARS-CoV-2 VLP production and the effect of S mutation on VLP assembly. Plasmids encoding SARS-CoV-2 structural proteins E, M, and N were cotransfected in HEK293T cells with plasmids encoding the WT S or its mutants. VLPs were enriched from culture media at 72 h posttransfection by ultracentrifugation. The mutation of L753, Y756, F759, or R815 affects the incorporation of S protein into SARS-CoV-2 VLPs and the generation of the complete VLPs.

The next question is whether the above-mentioned locus mutations affect the production of authentic SARS-CoV-2 virions. The SARS-CoV-2 virion, as is well established, is composed of four structural proteins: spike, membrane, envelope, and nucleocapsid protein. Recent studies have shown that the coexpression of four structural proteins in HEK293T cells could result in self-assembly into virus-like particles (VLPs) (25, 28, 29). Therefore, the effect of Y756 mutation on incorporation of S protein into virion particles was assessed using the virus-like particle production system. The expression of the four structural proteins in cotransfected cells or the pellet after ultracentrifugation of cotransfected cell culture supernatant was detected by Western blotting. In the VLP-producing cells (Fig. 6A), the expression of the WT S and its mutants was consistent with that previously described for pseudovirus-producing cells (Fig. 4A). Furthermore, the Western blot results with antibody targeting S2 showed that the Y756 mutation directly interfered with the processing of the S protein into S1, S2, or S2′ (Fig. 6A), especially when Y756 was mutated to cysteine, alanine, glycine, or glutamate. The results suggest that Y756 is critical for recognition of the S1/S2 and/or S2′ site by corresponding proteolytic enzymes. Furthermore, the M, N, and E proteins were all detectable in the cell lysate (Fig. 6A).

For the VLPs, the WT or D614G mutated S proteins incorporated into SARS-CoV-2 VLPs composed of M, E, and N (Fig. 6B). However, unlike the former two, part of the S proteins was incorporated into VLPs in the noncleavage form when R685 was mutated to A in the S1/S2 cleavage site (Fig. 6B). In contrast, in the case of the R815 mutation, VLPs containing four structural proteins were not detectable in the pellet, and the S, M, and E proteins were not detectable in the pellet (Fig. 6B). The above-described results indicate that mutation of the S2′ site leads to the failure of VLP assembly, but S1/S2 site mutation does not, and the S2′ site is also critical for incorporating S protein into VLPs and assembly of virion particles. Coincidentally, Y756 and R815 are functionally similar. The Y756C, Y756A, Y756G, and Y756E S mutants were weakly observed as a noncleavage form in the pellets, and the E protein was not detectable in these pellets (Fig. 6B). In addition, when Y756 was mutated to F, W, or H, the S proteins were incorporated into VLPs at lower levels than the WT or D614G mutated S proteins (Fig. 6B). These data indicate that residue Y756 in the S2 subunit is important for forming SARS-CoV-2 VLPs. L753, F759, and T998, structurally related to Y756, L753G, F759G, and T998G mutants, and N and M proteins in the producer cells were detected by Western blotting (Fig. 6C).

Furthermore, in the pellets, compared to VLPs with WT S protein, F759G or T998G mutant VLPs only contained lower levels of cleaved S1 or S2 products, especially F759G mutant, whereas L753G spike did not appear in the precipitation (Fig. 6C). These results illustrate that the amino acid residues linked to Y756 are closely related to the processing of S protein and the formation of virus-like particles. Together, our data suggest that the assembly and release of SARS-CoV-2 VLPs are weakened when the Y756-mutated spike could be cleaved. Nevertheless, the assembly and release of VLPs were inhibited when the Y756-mutated spike could not be cleaved (Fig. 6D). Similar to the R815 mutation, the Y756-associated L753 and F759 mutations also affect the incorporation of S protein into SARS-CoV-2 virus-like particles, suggesting that Y756 and associated residues are needed for efficient folding of the S protein.

### Effect of S mutation on neutralization potency of the antibodies targeting the receptor-binding domain.

**FIG 7:**
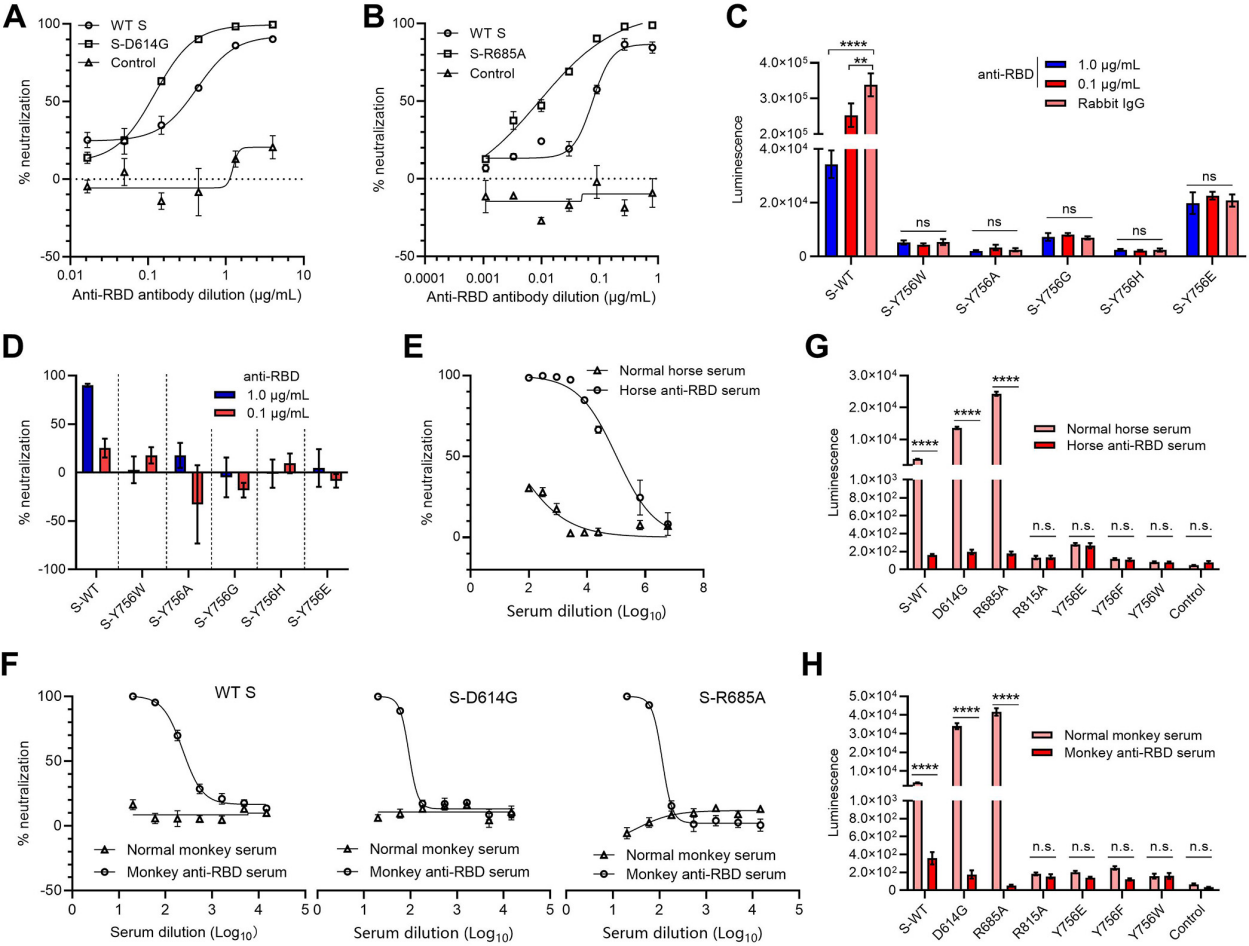
Effects of R685, Y756, or R815 mutations on the neutralization sensitivity of the antibodies targeting the receptor-binding domain of S protein. (A and B) The lentiviruses pseudotyped with WT, D614G (A), or R685A (B) mutant S proteins were preincubated with 3-fold serially diluted anti-RBD monoclonal antibody and then infected 293T-hACE2 cells for 48 h, and luciferase assays determined the infection levels of pseudoviruses in the presence of diluted antibodies. VSV G-incorporated pseudotyped viruses were used as a nonspecific control. (C and D) Neutralization sensitivity (C) and potency (D) analysis of Y756W, Y756A, Y756G, Y756H, or Y756E mutant S pseudotyped viruses with the indicated concentration of anti-RBD monoclonal antibody by luciferase activity assays. (E and F) Neutralization potency of horse (E) or monkey (F) anti-RBD serum obtained from a horse or monkey immunized with RBD against SARS-CoV-2 S pseudotyped viruses. (G) Analysis of neutralization sensitivity of R685A, R815A, or Y756 mutant S pseudotyped viruses to the indicated concentration of horse anti-RBD serum by luciferase activity assays. (H) Analysis of neutralization sensitivity of R685A, R815A, or Y756X mutant S pseudotyped viruses to the indicated concentration of monkey anti-RBD serum by luciferase activity assays, X represents the specified amino acid. Data are presented as means ± SD from 6 replicates. **, *P < *0.01; ****, *P < *0.0001 (Student *t* test). Data are representative of those from three independent experiments.

The above-described results confirmed that although the R685A mutation causes the S protein to be incompletely cleaved, it can be assembled into pseudovirions with the typical spike structure of the SARS-CoV-2 prototypic virus. So then, an important question is raised: does this mutation affect antiviral therapy targeting the S protein? The neutralization potency of the monoclonal antibodies and horse or rhesus monkey antiserum targeting the S protein receptor-binding domain was assessed using the pseudotyped viruses bearing the WT S or D614G variant and vesicular stomatitis virus G protein (VSV G)-enveloped pseudotyped virus as a nonspecific control. As expected, the anti-RBD monoclonal antibodies efficiently neutralized both viruses pseudotyped with the WT S and D614G variant but not VSV G-enveloped pseudotyped viruses (Fig. 7A), which was consistent with the previous results of the neutralization assays using the SARS-CoV-2-infected patient serum (25, 30). The results indicated that the anti-RBD monoclonal antibodies showed neutralization potency against SARS-CoV-2 S pseudoviruses at certain concentrations, and it had neutralizing activity to the D614G variant. Also, the R685A mutation did not affect neutralization mediated by anti-RBD monoclonal antibodies (Fig. 7B). In addition, although Y756 mutations seriously interfere with the incorporation of S protein into virus particles, the pseudoviruses bearing Y756E mutant S generate a slightly significant luciferase signal. So to evaluate the effect of the Y756E mutation on the antigenicity of the S protein, neutralization experiments were carried out; they showed that the pseudotyped viruses lose neutralization sensitivity to the anti-RBD monoclonal antibody (Fig. 7C and D). Then, neutralization assays were performed to assess the neutralization sensitivity of the R685A and Y756E mutants to horse or monkey anti-RBD serum compared to the WT S protein using the unimmunized donor serum as a negative control. Both anti-RBD sera demonstrated high neutralization titers against the pseudoviruses with WT SARS-CoV-2 S and the D614G or R685A mutant (Fig. 7E and F), and the Y756 mutation and R815 mutation but not R685A made the sensitivity of neutralizing antibody against SARS-CoV-2 S disappear (Fig. 7G and H). In summary, Y756 and R815 in the SARS-CoV-2 S protein are important for production, transduction, infectivity, and antigenicity of pseudotyped viruses.

## DISCUSSION

At the end of 2019, severe acute respiratory syndrome coronavirus 2 (SARS-CoV-2) as a novel betacoronavirus broke into people’s lives; this virus had highly transmissible and pathogenic characteristics and caused a pandemic of acute respiratory disease named coronavirus disease 2019 (COVID-19) (31, 32). As we all know, the global spread and epidemic of SARS-CoV-2 have caused great trauma to society, economic development, and human life safety. Fortunately, the successful development and application of vaccines, including the inactivated viruses (33, 34), recombinant viral vector vaccines (35, 36), mRNA nucleic acid vaccines (37), and protein subunit vaccines (38), have contributed to prevention and control of SARS-CoV-2. However, the continuous emergence of virus variants, such as the B.1.1.7 strain in the United Kingdom, the B.1.351 strain in South Africa, the B.1.1.28.1 strain in Brazil, and the B.1.617 strain in India, not only caused the continuation of the epidemic but also challenged the effectiveness of these existing vaccines (39–41), especially the mutations of some amino acids in the spike protein. In these mutations, a natural D614G spike mutation has been demonstrated to increase viral infectivity and transmissibility (24, 25, 30, 42, 43) but does not affect neutralization sensitivity to antisera against prototypic viruses or anti-RBD monoclonal antibodies (25, 30), which is consistent with the results in our study (Fig. 7). Furthermore, in this study, we paid more attention to the effect of the two cleavage sites of S protein and the region between them on virus entry mediated by S protein.

The SARS-CoV-2 virion mainly consists of four structural proteins, including the spike, envelope (E), membrane (M), and nucleocapsid (N) proteins. For SARS-related coronaviruses, the S protein is synthesized and processed, including posttranslational modification and cleavage, in the endoplasmic reticulum (ER) and Golgi, respectively (44, 45). Moreover, in the ER-Golgi intermediate compartment (ERGIC), the coronavirus-like particles are assembled under the cooperation and interaction of four structural proteins (29). As the primary receptor binding and membrane fusion mediator, S protein is required for progeny viral particles to enter and infect cells successfully (28, 46). In prototypic virus particles (4, 20), VLPs (28, 46), or pseudovirus particles in our study (Fig. 4) and other studies (19, 26, 47), the S protein appeared as two forms: cleavage and noncleavage. Cleavage of the S protein at the S1/S2 (RRAR↓S motif) and/or S2′ site (PSKR↓S motif) is associated with viral entry, which can occur at either the cell plasma membrane (TMPRSS2 pathway) or the endosomal membrane (cathepsin L pathway), depending on the cell type (23, 48).

Moreover, other proteases’ S1/S2 cleavage is processed beyond the furin proteolytic enzyme (48). For SARS-CoV-2 S protein, we found that both mutations of R685 in the S1/S2 cleavage site and R815 in the S2′ site significantly reduce cleavage efficiency (Fig. 1E), but they have two completely different outcomes. The mutation of R685A does not alter S-mediated viral entry by pseudovirus, but the R815A mutation significantly affects S-mediated viral entry (Fig. 2). These results suggested that the cleavage of the spike between the S1 and S2 subunits can also occur, but S1/S2 cleavage does not appear to be essential for entry, which has been proved in furin knockout cells (49) and may explain the fact that the inhibitors targeting furin do not completely prevent viral infection. Furthermore, the TMPRSS2-recognized S2′ site is extraordinarily critical for SARS-CoV-2 S-mediated entry and fusion and is an important target for developing effective antiviral drugs (50).

The S2 subunit consists of the fusion peptide (FP), a second proteolytic site (S2′), an internal fusion peptide (IFP), and two heptad-repeat domains (HR1 and HR2) preceding the transmembrane domain (TM) (3, 45). Furthermore, the HR1 and HR2 domains interact to form a six-helix bundle (6-HB) fusion core after S-receptor binding, bringing viral and cellular membranes into proximity for fusion and infection (51). The spike protein must be cleaved at the S1/S2 and S2′ sites to catalyze the membrane fusion reaction (51, 52). However, the role of the conservative region between the S1/S2 and S2′ sites (about 130 amino acid residues) in the structure and function of the S protein is rarely reported. In this study, we have noticed that a tyrosine residue, Y756, in the region between the S1/S2 and S2′ sites could form three hydrogen bonds with L753, F759, and T998, which can be observed in the cryo-electron microscopy (cryo-EM) structure of the prefusion S protein (PDB code 6VSB) (6). Accordingly, we speculate that the destruction of the hydrogen bond formed by Y756 and L753, F759, or T998 would affect the structural stability of the S protein and its mediated virus entry. As expected, the mutation of Y756 to different amino acids caused the S protein to show two different expression patterns: cleavage or noncleavage (Fig. 1E and F). From the results, when Y756 was mutated to amino acid residues meeting the conditions for hydrogen bond formation, S protein could be expressed as a cleaved form, but not vice versa.

It is noteworthy that the changes in the expression and subcellular localization of the S protein affect the assembly of virus particles and viral transduction to a certain extent. Indeed, we observed very low or even no infectivity of Y756-mutated S-based pseudovirus compared to that with WT S protein under the conditions of our experiments (Fig. 2E), which may be related to the yield and quality of pseudotyped virus in producer cells. As expected, we also found that the Y756 mutation affected the packaging and viral morphology of the pseudotyped virus embedded in the S protein based on qualitative or semiquantitative analysis of S protein and P protein of HIV in culture supernatant containing pseudotyped viruses (Fig. 4B and C). Furthermore, negative-stain transmission electron microscopy was used to demonstrate that no significant spikes appeared on the surface of the pseudoviruses formed by Y756 mutant S, compared with that of the pseudoviruses bearing the WT S, D614G mutant S, or R685A mutant S. Therefore, the neutralization sensitivity of the pseudoviruses with Y756 variants to anti-RBD antibody or antiserum was altered in this work. Although there is no systematic report on the effect of Y756 mutation on the infectivity of infectious viruses at present, refer to the series of studies in which D614G was proved to be an infection-enhancing mutation in tissue culture using a pseudotyped virus system (24, 25, 30, 43, 53); the Y756 mutation in S protein may be infection attenuating, but this needs further confirmation with infectious virus. Follow-up research work is in progress in our group.

Taken together, our results show that the S protein of SARS-CoV-2 as the primary receptor binding and membrane fusion mediator mediates virus entry into host cells and is also one of the main structural proteins for virus assembly. Therefore, the correct expression, structural stability, and precise subcellular localization of the S protein are critical to the correct assembly of the virus and indirectly affect the infection of the progeny virus. In this work, we confirm for the first time that the tyrosine residue at position 756 and the associated L753 and F759 are critical to the correct expression and subcellular localization of the S protein, regulate virion assembly and production, and, in turn, affect the S protein-mediated virus entry, using a pseudotyped virus or virus-like particle model. In addition to the resolved cryo-EM structure of the S protein, we found that the hydrogen bond formed by Y756 is also important for the stability of the S protein structure. In terms of significance, this study provides a new potential target for novel interventions, including antiviral drugs or CRISPR-mediated gene mutation therapy, by analyzing the key amino acid sites of the S protein. In the future, we will cooperate with other teams to carry out authentic SARS-CoV-2 verification and small-molecule drug screening research due to biosafety factors.

## MATERIALS AND METHODS

### Plasmid constructs.

The full-length SARS-CoV-2 spike (S) gene was synthesized (General Biol, China) according to the sequence of SARS-CoV-2 Wuhan-Hu-1 strain (54) (GenBank accession no. NC_045512.2) and then inserted into the eukaryotic expression vector pCMV-3×Flag-14 and fused with 3×Flag for expression. The S mutants, including D614G, R685A, R815A, and series mutation of Y756, L753, F759, or T998, were engineered and constructed by PCR with a Q5 site-directed mutagenesis kit (New England BioLabs [NEB], USA), using the S gene of the Wuhan-Hu-1 strain as the parent and WT sequence. The WT S gene and its mutants fused with EGFP at the C terminus were cloned into the pEGFP-N3 vector for subcellular localization assays. Human *ACE2* was amplified using pcDNA3.1-ACE2-Flag (GenBank accession no. NM_021804.1; Beyotime, China) as a template and cloned into pLenti 6.3/V5-DEST (Invitrogen, USA) via homologous recombination to construct a cell line stably expressing human angiotensin-converting enzyme 2 (ACE2). The lentiviral backbone plasmid pNL4-3.Luc.R-E- was provided by Ningyi Jin. For the coexpression of SARS-CoV-2 M and E proteins, the M-IRES-E fusion fragment was composed of the M and E genes fused with Flag tag in the N and C termini, respectively, and M and E were linked by the internal ribosome entry site (IRES) of encephalomyocarditis virus (EMCV). The pcDNA3.1-SARS-CoV-2-N-Myc was purchased from Beyotime (China). All plasmids were verified by DNA sequencing.

### Reagents and antibodies.

Rabbit monoclonal antibody against the SARS-CoV-2 receptor-binding domain (RBD) (no. 40592-R001), rabbit polyclonal antibody against SARS-CoV-2 nucleocapsid (no. 40588-T62) and spike S2 (no. 40590-T62), and HIV-1 p24 protein (group M, subtype B, strain 92418) antibody (no. 11695-T48) were purchased from Sino Biological (China). Rabbit polyclonal antibodies against Flag (DYKDDDDK) tag (no. 20543-1-AP), ACE2 (no. 21115-1-AP), GAPDH (no. 10494-1-AP), and β-actin (no. 20536-1-AP), and CoraLite 488-conjugated ACE2 monoclonal antibody (no. CL488-66699) were purchased from Proteintech (China). Horse anti-RBD/SARS-CoV-2 spike serum and cynomolgus monkey anti-RBD serum were gifts from Ningyi Jin.

### Cells and 293T-hACE2 stable cell lines.

Human lung epithelial (A549) cells were maintained in Ham’s F-12K (Kaighn’s) medium (Gibco, USA) or supplemented with 10% fetal bovine serum (FBS; Gibco, USA) and 1% penicillin-streptomycin (Solarbio, China). HEK293T, HEK293, and HeLa cells were maintained in Dulbecco’s modified Eagle medium (DMEM; HyClone, USA) containing 10% FBS and 1% penicillin-streptomycin. Human hepatoma (Huh7) cells were cultured in DMEM supplemented with 10% FBS, 1% GlutaMAX (Thermo, USA), and 1 mM sodium pyruvate (Gibco, USA). These cells were cultured at 37°C in a 5% CO_2_ incubator and kindly provided by Stem Cell Bank, Chinese Academy of Sciences. A HEK293T cell line with stable expression of human ACE2, named 293T-hACE2, was generated based on a lentivirus-mediated gene transduction system under the antibiotic pressure of blasticidin (InvivoGen, USA). The expression of hACE2 was examined by Western blotting with anti-ACE2 antibody, and the hACE2^+^ cell percentage was identified by flow cytometry.

### Western blot analysis.

Cells were lysed with a buffer of 20 mM Tris (pH 7.5), 150 mM NaCl, 1% Triton X-100, and EDTA-free protease inhibitor cocktail (Roche, Switzerland) and phenylmethylsulfonyl fluoride (PMSF; Beyotime, China). The extracted proteins were subjected to SDS-PAGE and then electrophoretically transferred to a nitrocellulose membrane (GE Healthcare, Germany), and protein bands were probed with the indicated primary antibodies.

### Pseudovirus production and viral entry assays.

A total of 5 × 10^6^ HEK293T cells in a 10-cm dish were cotransfected with 8 μg of S and its mutant expression plasmids pCMV-S-3×Flag including the WT, D614G, R685A, R815A, or Y756 mutant or L753G, F759G, or T998G and 8 μg of pNL4-3.Luc.R-E- using Lipofectamine 3000 (Thermo, USA) to prepare various SARS-CoV-2 S-pseudotyped lentiviral luciferase reporter viruses. Six hours posttransfection, the supernatants were replaced with fresh DMEM supplemented with 2% FBS. The supernatants containing the pseudoviruses were harvested 48 h after transfection and purified by filtration with a 0.45-μm filter. In accordance with the manufacturer’s protocol, the pseudoviruses were titrated with the Lenti-X reverse transcription-quantitative PCR (qRT-PCR) titration kit (TaKaRa, Japan). For SARS-CoV-2 S-pseudotyped virus entry assays, Huh7 or 293T-hACE2 cells were infected with a fixed dose of pseudoviruses for 48 h and then washed and lysed to detect luciferase signal with the ONE-Glo luciferase assay system (Promega, USA) according to the instructions.

### Analysis of incorporation of spike into pseudotyped virus particles.

The supernatants containing the pseudoviruses with SARS-CoV-2 S protein or its mutants were obtained as described above and then layered onto 20% (wt/vol) sucrose cushions and purified and concentrated by ultracentrifugation (35,000 rpm, 2 h, 4°C) using an Optima XPN-100 ultracentrifuge (Beckman Coulter). Furthermore, the pellets were resuspended in sterilized 1× phosphate-buffered saline (PBS) and prepared for SDS-PAGE and Western blot analysis using the corresponding primary antibodies against RBD, Flag, or HIV p24.

### Negative-stain imaging of transmission electron microscopy.

The resuspended pellets containing the pseudovirions obtained as described above were added to a glow-discharged 230-mesh copper grid covered with carbon-coated collodion film. After standing for several minutes, the excess liquid was absorbed with a filter paper from the edge of the copper mesh. Then, the copper mesh was stained with phosphotungstic acid (2.0% [wt/vol]; Solarbio, China) for 5 min and then sucked dry with a filter paper. Samples were analyzed with a JEM-1200EXII transmission electron microscope (JEOL).

### Analysis of subcellular localization.

Analysis of the subcellular localization of C-terminally EGFP-tagged S or its mutants in HeLa cells was done using a laser scanning confocal microscope (63×, oil). The tyrosine protein kinase Lck was used as a subcellular localization marker. Briefly, HeLa cells were cultured on glass coverslips to cotransfect with S or its mutant expression plasmids and Lck marker for 48 h, then fixed in 4% paraformaldehyde and PBS for 30 min, and then permeabilized with 0.25% Triton X-100 according to the specific requirement. The nuclei were stained with 4′,6-diamidino-2-phenylindole (DAPI; Invitrogen, USA). Images were taken under a fluorescence microscope or a Leica TCS SP8 confocal microscope.

### Production of SARS-CoV-2 VLPs.

HEK293T cells in a 10-cm dish were cotransfected with the plasmids encoding the SARS-CoV-2 M, E, N, and S or its mutants. In the transfection mixtures, 6 μg of each plasmid M-IRES-E, pcDNA3.1-SARS-CoV-2-N-Myc, and pCMV-3×Flag-S or the plasmid with S mutant were added into 1 mL of Opti-MEM and then mixed with 18 μL of PEIpro transfection reagent (Polyplus-transfection, France). The transfection mixtures were incubated for 15 min at room temperature and dropped into HEK293T cells. At 72 h posttransfection, the culture medium was collected, filtered through a 0.45-μm filter, layered onto 20% (wt/vol) sucrose cushions, and purified and concentrated by ultracentrifugation (35,000 rpm, 2 h, 4°C) using an Optima XPN-100 ultracentrifuge (Beckman Coulter) as described above. Moreover, the pellets containing VLPs were resuspended in sterilized 1× PBS, aliquoted, and stored at −80°C freezer.

### Composition analysis of SARS-CoV-2 VLPs.

The VLP pellets and the producer cell lysates were boiled with 5× SDS-PAGE loading buffer for 10 min at 100°C to identify the components of VLPs. The cell lysates were used to normalize loading conditions for SARS-CoV-2 structure proteins. These samples were resolved by a 12.5% SDS-PAGE separating gel and subsequently transferred to a nitrocellulose membrane for Western blot analysis with the indicated antibodies against SARS-CoV-2 S RBD S2 or N Flag tag. GAPDH was the loading control.

### Neutralization assays.

The SARS-CoV-2 pseudovirus neutralization assay was used to test the effect of mutation on S antigenicity. The horse or rhesus monkey anti-RBD serum samples collected after two immunizations were gifts from Ningyi Jin and were inactivated at 56°C for 30 min. 293T-hACE2 cells were seeded in 96-well tissue culture plates at a density of 1 × 10^4^/well overnight. Threefold serial dilutions of a commercially available rabbit anti-RBD monoclonal antibody or anti-RBD serum samples were prepared and mixed with an equal volume of pseudoviruses. The mixture was incubated at 37°C for 1 h before adding 293T-hACE2 cells. At 48 h after infection, cells were lysed to detect luciferase signal with the ONE-Glo luciferase assay system (Promega, USA) to determine the neutralization levels.

### Statistical analyses.

Data are presented as means ± standard deviations (SD) from 3 to 6 replicates. Data are representative of those from three independent experiments. Statistical analyses of the data were performed by using GraphPad Prism version 8.4.3. Statistical comparisons were made using a two-tailed paired or unpaired Student *t* test or one-way analysis of variance (ANOVA) with Dunnett’s multiple-comparison test.
