# Supplementary material for: The “LLQY” Motif on SARS-CoV-2 Spike Protein Affects S Incorporation into Virus Particles
Source: J Virol. 2022 Mar 23;96(6):e01897-21. doi: 10.1128/jvi.01897-21 (PMC8941915; doi:10.1128/jvi.01897-21)
Supplement: Supplemental file 1 — Fig. S1. Download jvi.01897-21-s0001.pdf, PDF file, 0.8 MB [file jvi.01897-21-s0001.pdf]

Supplemental materials

Figure S1: Sequence alignment analysis of the amino acids of SARS-CoV-2 Spike protein. (A) The conservation analysis of the full-length S proteins from 27 SARS-CoV-2 isolates from different countries or regions and S proteins from SARS-CoV CUHK-W1 strain or MERS-CoV HCoV-EMC/2012. (B) The conservation analysis of the region between the S1/S2 and S2' cleavage site of S protein in the amino acid levels.

Figure S1

A

Percent Identity

	1	2	3	4	5	6	7	8	9	10	11	12	13	14	15	16	17	18	19	20	21	22	23	24	25	26	27	28		
**1**		99.9	99.9	99.8	99.8	99.8	99.9	99.9	99.8	99.9	99.8	99.9	99.8	99.8	99.9	99.8	99.8	99.8	99.8	99.8	99.8	99.9	99.8	99.8	99.9	99.9	74.1	26.5	**1**	MN988668.1-China.seq
**2**	0.0		99.9	99.8	99.8	99.8	99.9	99.9	99.8	99.9	99.8	99.9	99.8	99.8	99.9	99.8	99.8	99.8	99.8	99.8	99.8	99.9	99.8	99.8	99.9	99.9	74.1	26.5	**2**	MN988669.1-China.seq
**3**	0.0	0.0		99.8	99.8	99.8	99.9	99.9	99.8	99.9	99.8	99.9	99.8	99.8	99.9	99.8	99.8	99.8	99.8	99.8	99.8	99.9	99.8	99.8	99.9	99.9	74.1	26.5	**3**	MT066176.1-China.seq
**4**	0.1	0.1	0.1		99.9	99.9	99.8	99.8	99.8	99.8	99.8	99.8	99.9	99.9	99.8	99.8	99.8	99.9	99.8	99.8	99.9	99.8	99.8	99.8	99.8	99.8	74.0	26.3	**4**	LC547522.1-Japan.seq
**5**	0.1	0.1	0.1	0.0		99.9	99.8	99.8	99.8	99.8	99.8	99.8	99.9	99.9	99.8	99.8	99.8	99.9	99.8	99.8	99.9	99.8	99.8	99.8	99.8	99.8	74.0	26.3	**5**	LC547530.1-Japan.seq
**6**	0.1	0.1	0.1	0.0	0.0		99.8	99.8	99.8	99.8	99.8	99.8	99.9	99.9	99.8	99.8	99.8	99.9	99.8	99.8	99.9	99.8	99.8	99.8	99.8	99.8	74.0	26.3	**6**	LC547533.1-Japan.seq
**7**	0.0	0.0	0.0	0.1	0.1	0.1		99.9	99.8	99.9	99.8	99.9	99.8	99.8	99.9	99.8	99.8	99.8	99.8	99.8	99.8	99.9	99.8	99.8	99.9	99.9	74.1	26.5	**7**	MT020881.1-USA.seq
**8**	0.0	0.0	0.0	0.1	0.1	0.1	0.0		99.8	99.9	99.8	99.9	99.8	99.8	99.9	99.8	99.8	99.8	99.8	99.8	99.8	99.9	99.8	99.8	99.9	99.9	74.1	26.5	**8**	MT039888.1-USA.seq
**9**	0.2	0.2	0.2	0.1	0.1	0.1	0.2	0.2		99.8	99.7	99.8	99.8	99.8	99.8	99.7	99.8	99.8	99.8	99.8	99.8	99.8	99.8	99.7	99.8	99.8	74.0	26.5	**9**	MT259281-USA.seq
**10**	0.0	0.0	0.0	0.1	0.1	0.1	0.0	0.0	0.2		99.8	99.9	99.8	99.8	99.9	99.8	99.8	99.8	99.8	99.8	99.8	99.9	99.8	99.8	99.9	99.9	74.1	26.5	**10**	MT126808-Brazil.seq
**11**	0.1	0.1	0.1	0.2	0.2	0.2	0.1	0.1	0.2	0.1		99.8	99.8	99.8	99.8	99.8	99.7	99.8	99.7	99.7	99.8	99.8	99.7	99.8	99.8	99.8	74.1	26.5	**11**	MT350282-Brazil.seq
**12**	0.0	0.0	0.0	0.1	0.1	0.1	0.0	0.0	0.2	0.0	0.1		99.8	99.8	99.9	99.8	99.8	99.8	99.8	99.8	99.8	99.9	99.8	99.8	99.9	99.9	74.1	26.5	**12**	MT358638-Germany.seq
**13**	0.1	0.1	0.1	0.0	0.0	0.0	0.1	0.1	0.1	0.1	0.2	0.1		99.9	99.8	99.8	99.8	99.9	99.8	99.8	99.9	99.8	99.8	99.8	99.8	99.8	74.0	26.3	**13**	MT358643-Germany.seq
**14**	0.1	0.1	0.1	0.0	0.0	0.0	0.1	0.1	0.1	0.1	0.2	0.1	0.0		99.8	99.8	99.8	99.9	99.8	99.8	99.9	99.8	99.8	99.8	99.8	99.8	74.0	26.3	**14**	MT270102-Germany.seq
**15**	0.0	0.0	0.0	0.1	0.1	0.1	0.0	0.0	0.2	0.0	0.1	0.0	0.1	0.1		99.8	99.8	99.8	99.8	99.8	99.8	99.9	99.8	99.8	99.9	99.9	74.1	26.5	**15**	MT451017-Australia.seq
**16**	0.1	0.1	0.1	0.2	0.2	0.2	0.1	0.1	0.2	0.1	0.2	0.1	0.2	0.2	0.1		99.7	99.8	99.7	99.7	99.8	99.8	99.7	99.8	99.8	99.8	74.1	26.5	**16**	MT007544.1-Australia.seq
**17**	0.2	0.2	0.2	0.1	0.1	0.1	0.2	0.2	0.2	0.2	0.2	0.2	0.1	0.1	0.2	0.2		99.8	99.8	99.8	99.8	99.8	99.8	99.7	99.8	99.8	74.0	26.5	**17**	MT451876-India.seq
**18**	0.1	0.1	0.1	0.0	0.0	0.0	0.1	0.1	0.1	0.1	0.2	0.1	0.0	0.0	0.1	0.2	0.1		99.8	99.8	99.9	99.8	99.8	99.8	99.8	99.8	74.0	26.3	**18**	MT509499-India.seq
**19**	0.2	0.2	0.2	0.1	0.1	0.1	0.2	0.2	0.2	0.2	0.2	0.2	0.1	0.1	0.2	0.2	0.2	0.1		99.8	99.8	99.8	99.8	99.7	99.8	99.8	74.1	26.3	**19**	MT496979-India.seq
**20**	0.2	0.2	0.2	0.1	0.1	0.1	0.2	0.2	0.2	0.2	0.2	0.2	0.1	0.1	0.2	0.2	0.2	0.1	0.2		99.8	99.8	99.8	99.7	99.8	99.8	74.0	26.5	**20**	MT451882-India.seq
**21**	0.1	0.1	0.1	0.0	0.0	0.0	0.1	0.1	0.1	0.1	0.2	0.1	0.0	0.0	0.1	0.2	0.1	0.0	0.1	0.1		99.8	99.8	99.8	99.8	99.8	74.0	26.3	**21**	MT470219-Colombia.seq
**22**	0.0	0.0	0.0	0.1	0.1	0.1	0.0	0.0	0.2	0.0	0.1	0.0	0.1	0.1	0.0	0.1	0.2	0.1	0.2	0.2	0.1		99.8	99.8	99.9	99.9	74.1	26.5	**22**	MT256924-Colombia.seq
**23**	0.2	0.2	0.2	0.1	0.1	0.1	0.2	0.2	0.2	0.2	0.2	0.2	0.1	0.1	0.2	0.2	0.2	0.1	0.2	0.2	0.1	0.2		99.7	99.8	99.8	74.0	26.5	**23**	MT459872-Greece.seq
**24**	0.1	0.1	0.1	0.2	0.2	0.2	0.1	0.1	0.2	0.1	0.2	0.1	0.2	0.2	0.1	0.2	0.2	0.2	0.2	0.2	0.2	0.1	0.2		99.8	99.8	74.0	26.5	**24**	MT470104-France.seq
**25**	0.0	0.0	0.0	0.1	0.1	0.1	0.0	0.0	0.2	0.0	0.1	0.0	0.1	0.1	0.0	0.1	0.2	0.1	0.2	0.2	0.1	0.0	0.2	0.1		99.9	74.1	26.5	**25**	MT077125.1-Italy.seq
**26**	0.0	0.0	0.0	0.1	0.1	0.1	0.0	0.0	0.2	0.0	0.1	0.0	0.1	0.1	0.0	0.1	0.2	0.1	0.2	0.2	0.1	0.0	0.2	0.1	0.0		74.1	26.5	**26**	MT072688.1-Nepal.seq
**27**	26.3	26.3	26.3	26.5	26.5	26.5	26.3	26.3	26.5	26.3	26.3	26.3	26.5	26.5	26.3	26.3	26.6	26.5	26.3	26.6	26.5	26.3	26.6	26.5	26.3	26.3		25.9	**27**	SARS.seq
**28**	144.1	144.1	144.1	144.1	144.1	144.1	144.1	144.1	144.1	144.1	144.1	144.1	144.1	144.1	144.1	144.6	144.1	144.1	144.1	144.6	144.1	144.1	144.6	144.6	144.1	144.1	142.9		**28**	MERS.seq
	**1**	**2**	**3**	**4**	**5**	**6**	**7**	**8**	**9**	**10**	**11**	**12**	**13**	**14**	**15**	**16**	**17**	**18**	**19**	**20**	**21**	**22**	**23**	**24**	**25**	**26**	**27**	**28**		

B

Percent Identity

	1	2	3	4	5	6	7	8	9	10	11	12	13	14	15	16	17	18	19	20	21	22	23	24	25	26	27	28		
**1**		100.0	100.0	100.0	100.0	100.0	100.0	100.0	100.0	100.0	100.0	100.0	100.0	100.0	100.0	100.0	100.0	100.0	100.0	100.0	100.0	100.0	100.0	100.0	100.0	100.0	80.0	30.0	**1**	MN988668.1-China.seq
**2**	0.0		100.0	100.0	100.0	100.0	100.0	100.0	100.0	100.0	100.0	100.0	100.0	100.0	100.0	100.0	100.0	100.0	100.0	100.0	100.0	100.0	100.0	100.0	100.0	100.0	80.0	30.0	**2**	MN988669.1-China.seq
**3**	0.0	0.0		100.0	100.0	100.0	100.0	100.0	100.0	100.0	100.0	100.0	100.0	100.0	100.0	100.0	100.0	100.0	100.0	100.0	100.0	100.0	100.0	100.0	100.0	100.0	80.0	30.0	**3**	MT066176.1-China.seq
**4**	0.0	0.0	0.0		100.0	100.0	100.0	100.0	100.0	100.0	100.0	100.0	100.0	100.0	100.0	100.0	100.0	100.0	100.0	100.0	100.0	100.0	100.0	100.0	100.0	100.0	80.0	30.0	**4**	LC547522.1-Japan.seq
**5**	0.0	0.0	0.0	0.0		100.0	100.0	100.0	100.0	100.0	100.0	100.0	100.0	100.0	100.0	100.0	100.0	100.0	100.0	100.0	100.0	100.0	100.0	100.0	100.0	100.0	80.0	30.0	**5**	LC547530.1-Japan.seq
**6**	0.0	0.0	0.0	0.0	0.0		100.0	100.0	100.0	100.0	100.0	100.0	100.0	100.0	100.0	100.0	100.0	100.0	100.0	100.0	100.0	100.0	100.0	100.0	100.0	100.0	80.0	30.0	**6**	LC547533.1-Japan.seq
**7**	0.0	0.0	0.0	0.0	0.0	0.0		100.0	100.0	100.0	100.0	100.0	100.0	100.0	100.0	100.0	100.0	100.0	100.0	100.0	100.0	100.0	100.0	100.0	100.0	100.0	80.0	30.0	**7**	MT020881.1-USA.seq
**8**	0.0	0.0	0.0	0.0	0.0	0.0	0.0		100.0	100.0	100.0	100.0	100.0	100.0	100.0	100.0	100.0	100.0	100.0	100.0	100.0	100.0	100.0	100.0	100.0	100.0	80.0	30.0	**8**	MT039888.1-USA.seq
**9**	0.0	0.0	0.0	0.0	0.0	0.0	0.0	0.0		100.0	100.0	100.0	100.0	100.0	100.0	100.0	100.0	100.0	100.0	100.0	100.0	100.0	100.0	100.0	100.0	100.0	80.0	30.0	**9**	MT259281-USA.seq
**10**	0.0	0.0	0.0	0.0	0.0	0.0	0.0	0.0	0.0		100.0	100.0	100.0	100.0	100.0	100.0	100.0	100.0	100.0	100.0	100.0	100.0	100.0	100.0	100.0	100.0	80.0	30.0	**10**	MT126808-Brazil.seq
**11**	0.0	0.0	0.0	0.0	0.0	0.0	0.0	0.0	0.0	0.0		100.0	100.0	100.0	100.0	100.0	100.0	100.0	100.0	100.0	100.0	100.0	100.0	100.0	100.0	100.0	80.0	30.0	**11**	MT350282-Brazil.seq
**12**	0.0	0.0	0.0	0.0	0.0	0.0	0.0	0.0	0.0	0.0	0.0		100.0	100.0	100.0	100.0	100.0	100.0	100.0	100.0	100.0	100.0	100.0	100.0	100.0	100.0	80.0	30.0	**12**	MT358638-Germany.seq
**13**	0.0	0.0	0.0	0.0	0.0	0.0	0.0	0.0	0.0	0.0	0.0	0.0		100.0	100.0	100.0	100.0	100.0	100.0	100.0	100.0	100.0	100.0	100.0	100.0	100.0	80.0	30.0	**13**	MT358643-Germany.seq
**14**	0.0	0.0	0.0	0.0	0.0	0.0	0.0	0.0	0.0	0.0	0.0	0.0	0.0		100.0	100.0	100.0	100.0	100.0	100.0	100.0	100.0	100.0	100.0	100.0	100.0	80.0	30.0	**14**	MT270102-Germany.seq
**15**	0.0	0.0	0.0	0.0	0.0	0.0	0.0	0.0	0.0	0.0	0.0	0.0	0.0	0.0		100.0	100.0	100.0	100.0	100.0	100.0	100.0	100.0	100.0	100.0	100.0	80.0	30.0	**15**	MT451017-Australia.seq
**16**	0.0	0.0	0.0	0.0	0.0	0.0	0.0	0.0	0.0	0.0	0.0	0.0	0.0	0.0	0.0		100.0	100.0	100.0	100.0	100.0	100.0	100.0	100.0	100.0	100.0	80.0	30.0	**16**	MT007544.1-Australia.seq
**17**	0.0	0.0	0.0	0.0	0.0	0.0	0.0	0.0	0.0	0.0	0.0	0.0	0.0	0.0	0.0	0.0		100.0	100.0	100.0	100.0	100.0	100.0	100.0	100.0	100.0	80.0	30.0	**17**	MT451876-India.seq
**18**	0.0	0.0	0.0	0.0	0.0	0.0	0.0	0.0	0.0	0.0	0.0	0.0	0.0	0.0	0.0	0.0	0.0		100.0	100.0	100.0	100.0	100.0	100.0	100.0	100.0	80.0	30.0	**18**	MT509499-India.seq
**19**	0.0	0.0	0.0	0.0	0.0	0.0	0.0	0.0	0.0	0.0	0.0	0.0	0.0	0.0	0.0	0.0	0.0	0.0		100.0	100.0	100.0	100.0	100.0	100.0	100.0	80.0	30.0	**19**	MT496979-India.seq
**20**	0.0	0.0	0.0	0.0	0.0	0.0	0.0	0.0	0.0	0.0	0.0	0.0	0.0	0.0	0.0	0.0	0.0	0.0	0.0		100.0	100.0	100.0	100.0	100.0	100.0	80.0	30.0	**20**	MT451882-India.seq
**21**	0.0	0.0	0.0	0.0	0.0	0.0	0.0	0.0	0.0	0.0	0.0	0.0	0.0	0.0	0.0	0.0	0.0	0.0	0.0	0.0		100.0	100.0	100.0	100.0	100.0	80.0	30.0	**21**	MT470219-Colombia.seq
**22**	0.0	0.0	0.0	0.0	0.0	0.0	0.0	0.0	0.0	0.0	0.0	0.0	0.0	0.0	0.0	0.0	0.0	0.0	0.0	0.0	0.0		100.0	100.0	100.0	100.0	80.0	30.0	**22**	MT256924-Colombia.seq
**23**	0.0	0.0	0.0	0.0	0.0	0.0	0.0	0.0	0.0	0.0	0.0	0.0	0.0	0.0	0.0	0.0	0.0	0.0	0.0	0.0	0.0	0.0		100.0	100.0	100.0	80.0	30.0	**23**	MT459872-Greece.seq
**24**	0.0	0.0	0.0	0.0	0.0	0.0	0.0	0.0	0.0	0.0	0.0	0.0	0.0	0.0	0.0	0.0	0.0	0.0	0.0	0.0	0.0	0.0	0.0		100.0	100.0	80.0	30.0	**24**	MT470104-France.seq
**25**	0.0	0.0	0.0	0.0	0.0	0.0	0.0	0.0	0.0	0.0	0.0	0.0	0.0	0.0	0.0	0.0	0.0	0.0	0.0	0.0	0.0	0.0	0.0	0.0		100.0	80.0	30.0	**25**	MT077125.1-Italy.seq
**26**	0.0	0.0	0.0	0.0	0.0	0.0	0.0	0.0	0.0	0.0	0.0	0.0	0.0	0.0	0.0	0.0	0.0	0.0	0.0	0.0	0.0	0.0	0.0	0.0	0.0		80.0	30.0	**26**	MT072688.1-Nepal.seq
**27**	23.3	23.3	23.3	23.3	23.3	23.3	23.3	23.3	23.3	23.3	23.3	23.3	23.3	23.3	23.3	23.3	23.3	23.3	23.3	23.3	23.3	23.3	23.3	23.3	23.3	23.3		30.0	**27**	SARS.seq
**28**	127.0	127.0	127.0	127.0	127.0	127.0	127.0	127.0	127.0	127.0	127.0	127.0	127.0	127.0	127.0	127.0	127.0	127.0	127.0	127.0	127.0	127.0	127.0	127.0	127.0	127.0	134.3		**28**	MERS.seq
	**1**	**2**	**3**	**4**	**5**	**6**	**7**	**8**	**9**	**10**	**11**	**12**	**13**	**14**	**15**	**16**	**17**	**18**	**19**	**20**	**21**	**22**	**23**	**24**	**25**	**26**	**27**	**28**		
